# Schimmelpenning-Feuerstein-Mims syndrome: a systematic review of clinical cases to identify genotype-phenotype associations

**DOI:** 10.3389/fmed.2025.1681584

**Published:** 2025-12-08

**Authors:** Ekaterina Zelenova, Tatiana Belysheva, Elena Sharapova, Vera Semenova, Timur Valiev, Tatiana Nasedkina

**Affiliations:** 1 N.N. Blokhin National Medical Research Center of Oncology, Ministry of Health of the Russian Federation, Moscow, Russia; 2 Engelhardt Institute of Molecular Biology, Russian Academy of Sciences, Moscow, Russia; 3 Administrative Department of the President of Russia, Central State Medical Academy, Moscow, Russia; 4 I.M. Sechenov First Moscow State Medical University (Sechenov University), Moscow, Russia; 5 Dmitry Rogachev National Medical Research Center of Pediatric Hematology, Oncology and Immunology, Moscow, Russia

**Keywords:** Schimmelpenning-Feuerstein-Mims syndrome, *KRAS*, *HRAS*, *NRAS*, mutation, phenotype, mosaicism, dermal nevus

## Abstract

**Background/objectives:**

Schimmelpenning-Feuerstein-Mims syndrome (SFMS) is a rare genodermatosis from the group of epidermal nevus syndromes with a wide range of clinical manifestations, including linear nevus sebaceous, ophthalmological disorders, cardiovascular abnormalities, nervous system and musculoskeletal system lesions. Benign and malignant neoplasms, precocious puberty, lymphedema and malformations of the lymphatic system may also be present. The main cause of the syndrome are postzygotic mutations in the RAS pathway, namely in the *HRAS, KRAS*, and *NRAS* genes. The aim of our work was to analyze previously described and genetically confirmed clinical cases of SFMS in order to reveal genotype-phenotype correlations.

**Methods:**

A systematic literature review (SLR) was conducted in several databases, including PubMed and ResearchGate to search for clinical cases of SFMS with genetic testing data from 1946 to 2025. The criterion for inclusion was considered to be the reference to SFMS or its synonyms in article titles with no limitations on language or date of publication. Also, no restrictions on patient age, ethnicity, presence/absence of photo-materials were applied. Only original articles reporting clinical case or case series with detection of postzygotic mutations in the *HRAS*, *KRAS* or *NRAS* genes were included.

**Results:**

Currently, 50 patients with SFMS and identified pathogenic variants in the *HRAS* (*n* = 17), *KRAS* (*n* = 30), or *NRAS* (*n* = 3) genes have been described in the literature. Analyzing presented phenotypic features of genetically confirmed cases of SFMS depending on the affected gene, we found that ophthalmological and central nervous system anomalities were more common for the *KRAS-mut* patients (*p* < 0.05). In contrast, patients with *HRAS* mutations were more likely to have skeletal abnormalities (*p* < 0.05). Benign and malignant tumors were diagnosed with equal frequency in both genetic groups, but renal tumors were strongly associated with *KRAS* mutations.

**Conclusion:**

The data obtained indicates the necessity of genetic testing for all patients suspected of SFMS to determine the individual risk of comorbidities and to form a personalized plan for dynamic follow-up.

**Systematic review registration:**

https://www.crd.york.ac.uk/PROSPERO/view/CRD420251085872, identifier CRD420251085872.

## 1 Introduction

Schimmelpenning-Feuerstein-Mims syndrome (SFMS, Solomon syndrome, Schimmelpenning syndrome) is an orphan disease associated with pathogenic variants in the *HRAS, KRAS*, or *NRAS* genes in mosaic form (1, 2). Molecular genetic testing for patients with SFMS has become more accessible in recent years and includes DNA testing of affected tissues (nevus, benign and malignant tumors) by Sanger sequencing (3, 4), next-generation sequencing (5), including whole-exome sequencing (1, 6), or by using commercial test systems to analyze hot-spot mutations in above-mentioned genes (7).

The first description of SFMS by Gustav Schimmelpenning in 1957 (8) included such signs as nevus sebaceous of Jadassohn, neurological and ophthalmological disorders. Currently, the clinical understanding of this disease has expanded significantly. In addition to the classic triad of manifestations, the patients may present with anomalies of musculoskeletal (9), cardiovascular (10) and lymphatic systems (7, 11, 12), as well as precocious puberty (9, 13), benign (14, 15), and malignant neoplasms (16, 17).

At present, the diagnosis is established on the basis of phenotypic features and the detection of postzygotic variants in the *HRAS, KRAS*, or *NRAS* genes in the tissues involved in the pathological process. In the routine genetic test of peripheral venous blood or other unaffected tissues, pathogenic variants in these genes are usually not identified.

New approaches in the form of targeted therapies have been added recently to standard treatments for individual manifestations of SFMS. For example, Haller CN et al., 2024, demonstrate the efficacy of topical application of trametinib (MEK inhibitor) in a 20-month-old girl with SFMS and a mutation in the *KRAS* gene as a reduction in pruritus and plaque thinning (18). Clinical experience with oral trametinib for the treatment of seizure syndrome in a 14-month-old boy with SFMS associated with a mutation in the *KRAS* gene was presented by a research team from D’Onofrio et al. (19). The use of monoclonal antibodies to fibroblast growth factor 23 (FGF23) was described as a therapy for hypophosphatemic rickets in a patient with a postzygotic c.182A > G mutation in the *HRAS* gene (20). Sirolimus, an mTOR inhibitor, has been successfully used in the treatment of lymphorrhea and genital lymphangioma in SFMS associated with c.34G > A mosaic variant in the *HRAS* gene (11). This highlights the importance of genetic testing to identify a molecular target for therapy in patients suspected for SFMS.

Previous studies of patients with SFMS have focused on describing unique phenotypes, as the disease is extremely rare and clinically variable. Since the syndrome is associated with postzygotic mutations, it is suggested that the severity of clinical manifestations depends on the variant allele frequency. However, a systematic analysis of data from different clinical descriptions of the syndrome to search for genotype-phenotype correlations has not yet been performed. In this review, we systematized the data of 50 patients with genetically confirmed SFMS in order to identify associations between the spectrum of clinical manifestations and mutations in a particular gene. The data obtained can be used to develop gene-mediated dynamic observation and to predict the complications of the syndrome including the risk of malignancies.

## 2 Methodology

This SLR was performed according to the PRISMA 2020 statement (21). The protocol was recorded on the website PROSPERO, the identification number CRD420251085872.

### 2.1 Search strategy

The systematic literature review (SLR) was conducted in open databases (PubMed and ResearchGate). The following keywords were used for searching articles: “Schimmelpenning,” “Schimmelpenning-Feuerstein-Mims,” “Solomon syndrome,” “mosaic RAS pathology,” “mosaic phakomatosis,” “postzygotic/mosaic mutation in HRAS/KRAS/NRAS.” A total of 6,299 articles from 1946 to 5 July 2025 were included in the analysis. Of these, 46 articles were selected, describing a total of 50 patients with SFMS who underwent molecular genetic testing (Sanger sequencing, exome sequencing, or commercial PCR test systems). Patients with confirmed molecular diagnosis are described in detail in our systematic review (Supplementary Table 1).

### 2.2 Eligibility criteria/in- and exclusion criteria

We included clinical empirical studies reporting patients with SFMS having the results of genetic testing (the presence of mutation in *HRAS*, *KRAS* or *NRAS* genes). Schimmelpenning syndrome is one of the epidermal nevus syndromes (ENS), a large group of diseases with overlapping phenotypes. Some selected patients were diagnosed with CSHS or ENS, but detailed clinical data analysis revealed that these patients had all the necessary SFMS features: congenital craniofacial epidermal nevus, extracutaneous pathology and postzygotic mutations in the *HRAS*, *KRAS* or *NRAS* genes. After histological examination, in some cases a nevus sebaceous (a subtype of the epidermal nevus) was identified. The studies included did not have any limitations on date or language. Also, no restrictions on patient age, ethnicity, and presence/absence of photos were applied. Reviews and articles contained just investigation of nevus tissue were excluded. Besides the article was excluded in case of misdiagnosed patients (patients with pigmented keratotic phacomatosis, Proteus syndrome, Garcia-Hafner-Happle syndrome etc.) or in case of mutation in other genes, as well as wild type genotype of samples after comprehensive genetic testing. In addition, the study was excluded when the full text was not available.

### 2.3 Data extraction

Two authors (EZ and TB) independently searched the articles (analyzed titles and abstracts), that meet the predefined inclusion criteria. One author (EZ) had investigated the PubMed database, while another author (TB) had worked with Research Gate. These two databases of all retrieved articles were compared, removing double variants and records marked as ineligible by automation tools. As a result, one common database was formed. Then books, comments, reviews, molecular investigations of cells and nevus tissue were removed. The remaining full-text articles were then carefully evaluated against the established eligibility criteria. In the absence of access to the full text, the article was removed from the following investigations.

The data collected from the eligible studies encompassed various aspects, including publication year, study design, age and gender of participants, the distribution of affected areas, the presence of non-skin anomalies (neurological, ophthalmological, cardiovascular, endocrinological) and malignant tumors, laboratory (biochemical, DNA-testing) and instrumental findings. The main results of the study focused on the frequency of mutations in the genes of the RAS signaling pathway, various skin and non-skin manifestations, as well as the identification of rare pathologies such as benign and malignant neoplasms, early sexual development. Secondary outcomes encompassed the correlation between genetic findings (gene and type of mutation) and clinical features.

### 2.4 Assessment of risk of bias

The quality of the included articles describing clinical cases was assessed by two authors (EZ and TB) using the JBI Checklist for Case reports (Supplementary Table 2). Any inconsistencies revealed were discussed with a third author (TN) to find a solution. The articles having more than 5 points were included in the study. We did not exclude articles that lacked a treatment description, as the treatment was not always necessary for patients with this syndrome. Of the 45 clinical cases, 28 (62%) received the maximum score of 8 (Supplementary Figure 1), and one article was assessed as a cross-sectional study and received a score of 5. In addition, we used our own checklist for understanding the quality of patient’s diagnostics. This checklist consisted of nine distinct domains, each addressing specific aspects: (1) gender and age of patient, (2) result of genetic testing, (3) presence of nevus sebaceous of Jadassohn, (4) neurological abnormalities, (5) skeletal anomalies, (6) cardiovascular disorders, (7) ophthalmological anomalies (8), presence of benign and malignant neoplasms, and (9) endocrinological diseases. For each study, this tool was applied and data were categorized as «yes» (the article contained this information, 1 point), «no» (the article did not contain this information, 0 point), or not applicable (for example, the signs were not developed due to young age of the patient, 0 point). So, all articles had given a number of points and total score from 4 to 9 indicating high-quality studies. Finally, the SLR was checked using the JBI Checklist for SLR (22), by one author (TN). According to 11 control questions, all answers were «yes» (Supplementary Table 3).

## 3 Results

### 3.1 Study selection

**FIGURE 1:**
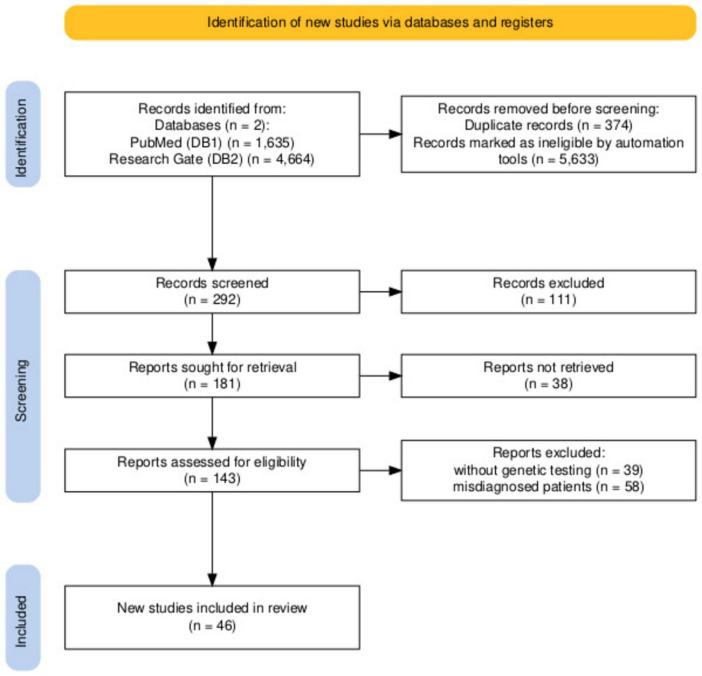
Search strategy.

In our systematic review, the search method yielded 6,299 records: 1,635 articles from the PubMed (database 1, DB1) and 4,664 articles from Research Gate (database 2, DB2), of which only 292 were retrieved after deleting duplicate articles (374) and articles from other topics (5,633), including the same name of authors, abbreviations etc. Then 111 articles (books, comments, reviews, histological studies of the nevi only) were removed. After full-text screening just 143 articles were appropriate for the next examination. Finally, we excluded 39 articles without genetic testing and 58 articles with misdiagnosed patients. So, the systematic review contained 46 papers, of which 37 were recognized as case reports, eight case series, and one as original study of nevus tissue from patients who was described earlier (Figure 1).

Patient ID19 was mentioned twice: for the first time as a case report (23) and for the second time in clinical series of cases after 1 year of observation (24). Patients ID40 and ID41 were also presented in three articles: The first record described phenotype and genetic damage, and other articles were devoted to the treatment of characteristic skeletal abnormalities (15, 25, 26). Patients ID48 and ID50 were described twice: first articles presented their clinical history as a case of SFMS (2, 9), and a later one published the results of genetic testing (3).

### 3.2 Demographic characteristic

In total, 50 patients with SFMS were identified and analyzed from 46 articles. The study sample was predominantly female (sex ratio 1,6:1). Thirteen patients were under 1 year of age, 12 patients were over 18 years of age, and the largest group was formed by patients aged from 1 to 18 years (25 patients). No one patient had familial history of this syndrome. Patient ID48 had a healthy twin brother (2, 3). Patients ID24 and ID33 were born to consanguineous marriage (20, 27). Two patients (ID22, ID48) died before 1 month of age due to combined malformations and respiratory impairment (2, 3, 24). Long-term follow-up was performed for patients ID15 (20 years), ID30 (>20 years), and ID31 (>20 years).

### 3.3 Genetic studies

**FIGURE 2:**
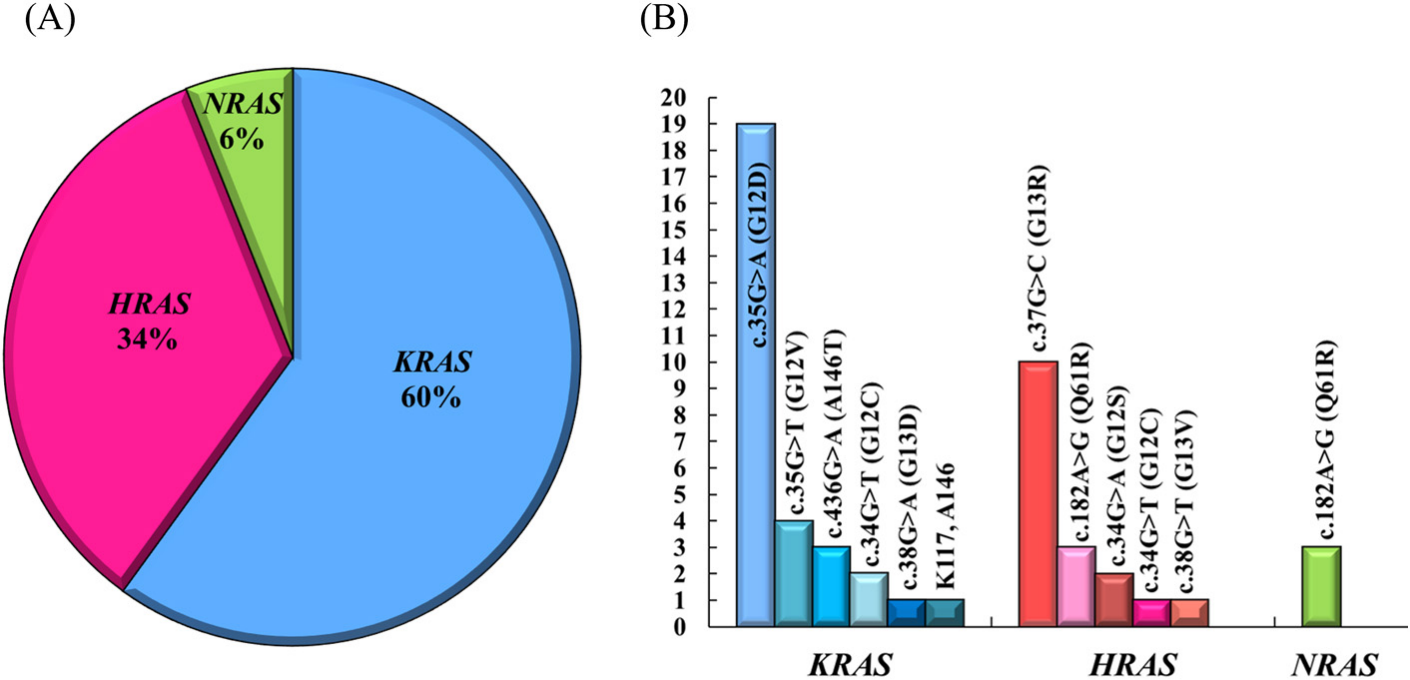
Spectrum and frequency of pathogenic variants in the *KRAS*, *HRAS* and *NRAS* genes in the patients with SFMS. (A) Distribution of mutations between the three genes. (B) Frequency of different nucleotide changes.

In all cases, genetic testing was performed (Sanger sequencing, exome sequencing or commercial PCR test systems). Out of 50 patients, pathogenic variants in the *KRAS, HRAS*, and *NRAS* genes were detected in 30, 17, and 3 patients, respectively (Figure 2). The most common pathogenic variant in the *KRAS* gene was c.35G > A (p. Gly12Asp or G12D), and in the *HRAS* gene c.37G > C (p.Gly13Arg or G13R). In all patients with affected *NRAS* gene a single nucleotide substitution c.182A > G (p.Gln61Arg or Q61R) was detected.

### 3.4 Diversity of clinical presentations of SFMS

All patients had a craniofacial linear epidermal nevus since birth. In the majority of articles histological examination was performed, and linear nevus sebaceous (type of epidermal nevus) was confirmed. Aplasia cutis has been reported in 3 cases, namely in patients ID3, ID22, and ID34 (24, 27, 28). Also, there were single cases of alopecia (29), bullous vesicles (28), hyperpigmentation (19, 24, 27, 30), café-au-lait spots (27, 31, 32), cellulitis progression to necrotizing fasciitis (24), giant Buschke-Lowenstein warts (11), basal cell carcinoma (3, 9, 32, 33), and syringocystadenoma papilliferum (9).

**FIGURE 3:**
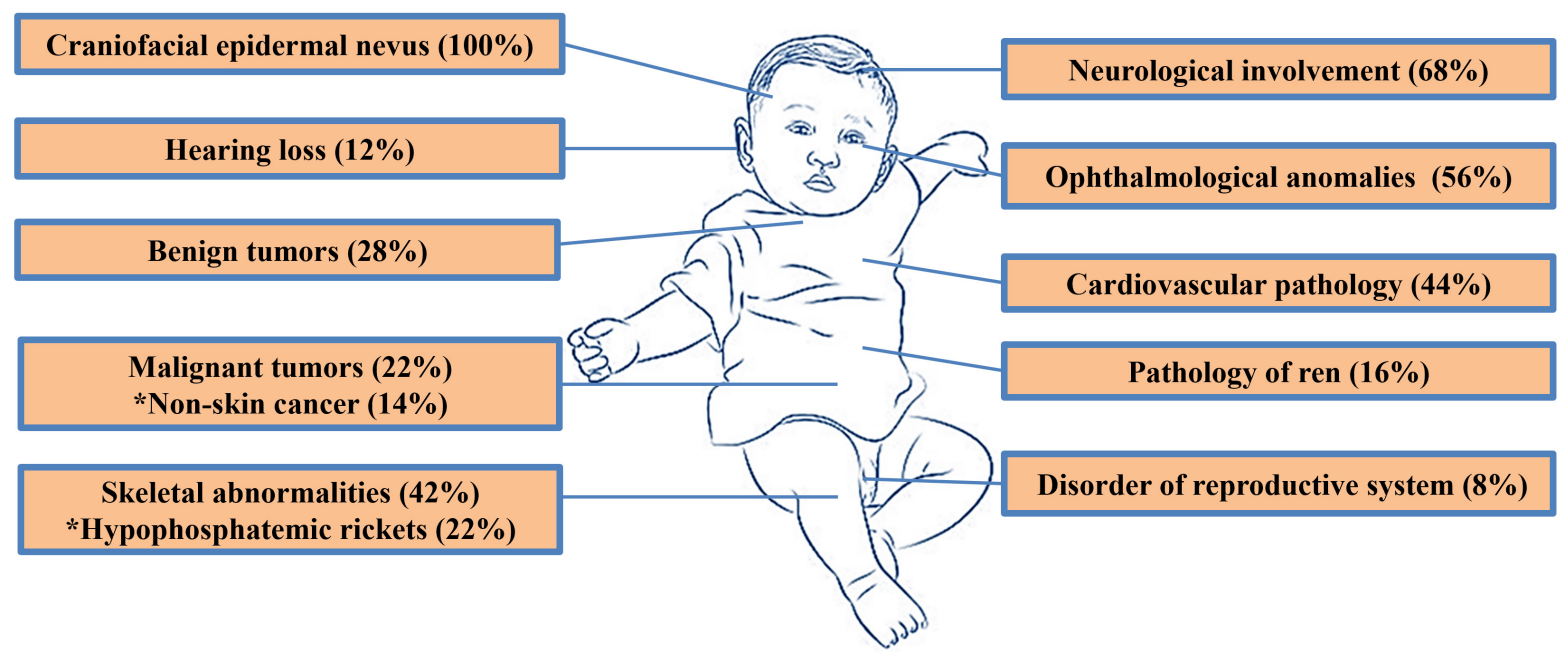
Distribution of phenotypic features in patients with SFMS presented in the current study.

The distribution of other prevailing manifestations was shown in Figure 3. Overall data about all patients from this study presented in Supplementary Table 1.

**FIGURE 4:**
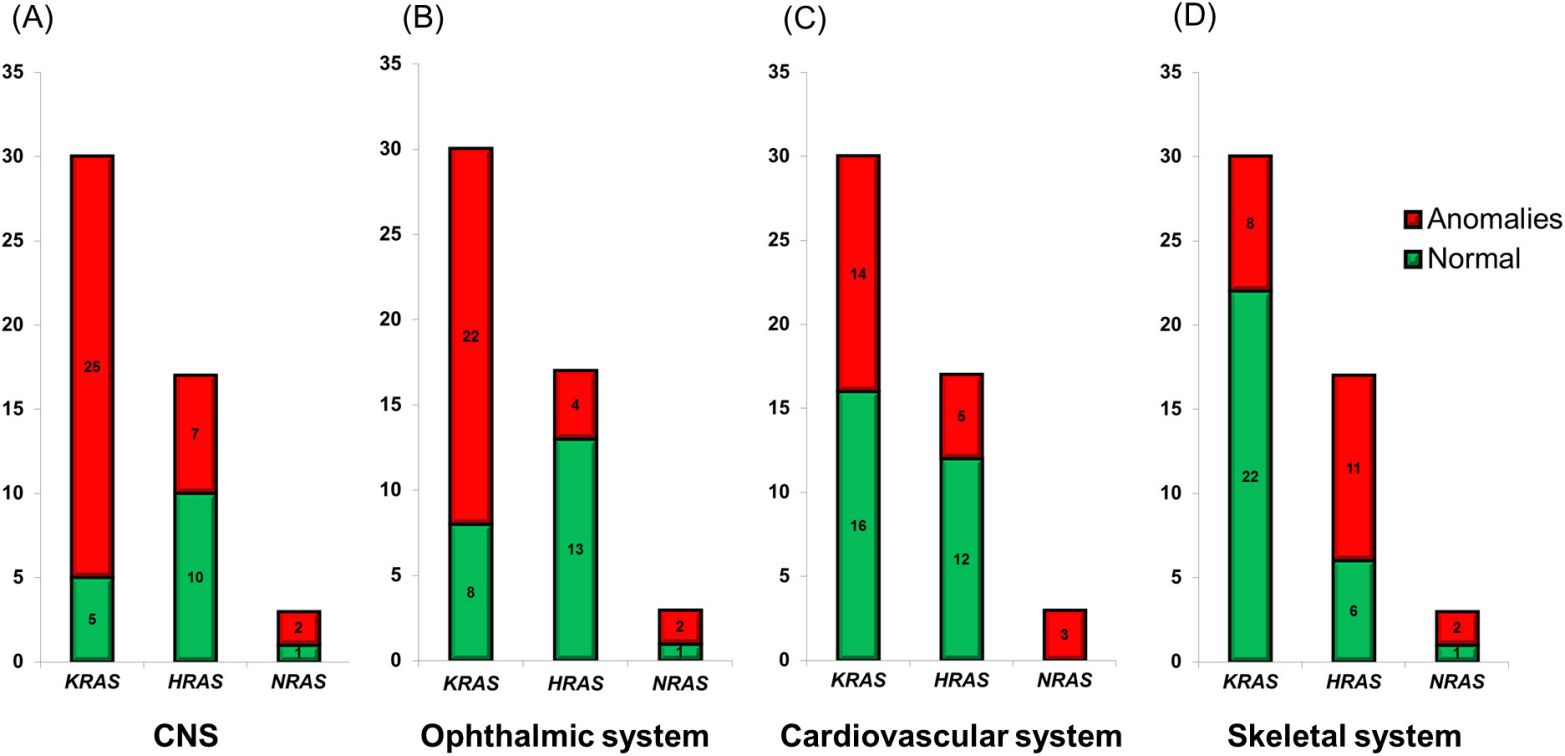
Clinical and genetic correlations of individual phenotypic manifestations of SFMS. (A) Anomalies of the central nervous system (CNS). (B) Ophthalmic anomalies. (C) Anomalies of cardiovascular system. (D) Skeletal abnormalities.

Central nervous system (CNS) abnormalities were diagnosed in 34 (68%) cases (Figure 3) and included epilepsy as the most common pathology (1, 5–7, 12, 14, 16, 18, 19, 24, 27, 30, 31, 34–40). Other disorders were represented by intellectual disability (5, 6, 12, 14, 16, 30, 38, 41), psychomotor delay (5, 7, 19, 37, 42), hemimegalencephaly (27, 40), macrogyria (1), polymicrogyria (27, 43), ventriculomegaly (1, 27), dysplasia of the brain (38), cortical atrophy (2, 3, 7, 12, 19, 27), hypotrophy of the hemisphere (43), cortical and hemisphere malformation (17, 35, 37), myelomeningocele (6), periventricular leukomalacia (24), paraplegia (6), abducens paresis (44), left-sided hypoglossal palsy (32), Chiari malformation (6), intracranial and intraspinal lipomas (15, 25, 28, 38, 40), focal subpial gliosis (5), hippocampal sclerosis (5), hippocampal asymmetry (14), interhemispheric asymmetry (27), arachnoid cyst (27, 42, 45, 46), spinal nerve root enlargement (34), thalamus and pineal hamartomas (17), and astrocytoma (47). Mutations in the *KRAS* gene were observed in most patients with CNS abnormalities (Figure 4A).

Ophtalmological lesions were also common among the patients with SFMS (Figure 3). They were described in 28 (56%) patients. Medical examination revealed reduced vision (48), chorioretinal and optic atrophy (29, 45), coloboma (2, 3, 5, 7, 17, 19, 27, 28, 40, 42), staphyloma (29), orbital choristoma (36, 43), epibulbar tumor (28), dermoid (1–3, 17, 27, 42, 46), limbal opacity (35), microcornea (2, 3), microphthalmia (17, 19), cryptophthalmos (2, 3), ptosis (19), Duane syndrome (19), ectropion (19), exotropia (31), sclerocornea (7, 19), amblyopia (38), dermolipoma (19), strabismus (9, 16, 44), choroidal osteoma (42), soft tissue tumor of the right lateral angle of the eye socket (31), brown spot on the conjunctiva (14), keratinisation of the conjunctiva (17), pterygium (12, 39), periorbital hamartoma (17), small macula (40), corneal clouding xanthelasmata (3, 9), closed eye slit on one side (1), optic disc abnormalities (5, 31), and lesion of the cornea (39). Mutations in the *KRAS* gene were also dominated in this group (Figure 4B).

Cardiovascular functional and structural anomalies were observed in 22 (44%) patients with SFMS (Figure 3). There was a wide spectrum of manifestations : vascular and heart capillary malformation (23, 24, 34, 40), hemangioma (15, 25, 40), lymphangioma (11, 43) and other vascular tumors (24), atrial septal defect (12, 20, 46), coarctation of the aorta (24), aneurysm of the interventricular septum (14), right aortic arch with aberrant left subclavian artery (19), aortic and subaortic valve stenosis (15, 25, 43), stenosis of the other artery (38), stroke in the middle right cerebral artery (6), patent ductus arteriosus (2, 3, 24), patent foramen ovale (16), abnormal tortuosity of the arteries (24), ectopic atrial tachycardia (17), cardiomegaly (17), chylothorax (24, 43), lymphangiectasia (24), lymphedema (7, 28), lymphatic dysplasia (16), and agenesis of the lymph nodes (7). These findings include lymphatic anomalies that were diagnosed in only seven patients (ID3, ID14, ID16, ID18, ID22, ID23 and ID37). The majority of patients with cardiovascular manifestations also had mutations in the *KRAS* gene (Figure 4C).

Skeletal abnormalities have been reported in 21 (42%) patients (Figure 3). The most common anomaly was hypophosphatemic rickets which was detected in 11 patients (ID2, ID14, ID24, ID30, ID31, ID32, ID38, ID40, ID41, ID43 and ID50). All of them had mutation in the *HRAS* (3, 9, 15, 20, 25, 32, 33, 41, 44, 49) or *NRAS* (15, 25, 43) genes. Other disorders included benign cortical defect of tibia (28), hexadactyly with a complete second thumb (4), polyostotic fibrous dysplasia (7), bone hyperplasia (16) or hypoplasia (31), osteosclerosis (23, 24), scoliosis (14), plagiocephaly (17), and maldevelopment of bone (12) and were associated with mutations in the *KRAS* (8 cases) or *HRAS* (2 cases) genes, respectively (Figure 4D).

Renal diseases were found in 8 (16%) patients with *KRAS* mutations (Figure 3). It was represented by polycystic kidneys (ID4, ID12, ID23, ID49) (19, 24, 34, 50), nephroblastomatosis (ID21) (24), bilateral Wilm’s tumor (ID19) (23, 24), kidney pelvic bifurcation (ID5) (35), and vascular renal hamartoma (ID36) (17).

Disorders of reproductive system were revealed in 4 (8%) patients (Figure 3). Three of these patients had a mutation in the *HRAS* gene (ID18, ID20 and ID50). In two cases (ID20 and ID50) it was a precocious sexual development (9, 13), while patient ID18 had anomalies of reproductive system in the form of cryptorchidism and hydrocele (16). The earliest age of manifestation of this endocrinopathy was 1 year and 5 months (ID20). Patient ID3 had a rare mutation c.436G > A in the *KRAS* gene and delayed puberty (28). Hearing loss was detected in 6 (12%) patients (Figure 3; 19, 24, 27, 32, 38, 48), most of these patients had mutations in the *KRAS* gene. Just in one case (ID8) of conductive loss of hearing (deformed malleus and incus) a pathogenic variant in the *HRAS* gene was detected (48).

Malignant tumors were reported in 11 (22%) patients (Figure 3). Four cases were presented by embryonal rhabdomyosarcoma (eRMS) of the inguinal region. One of these tumors (ID22) was congenital, the other neoplasia presented in patient ID49 at the age of 6 months, while in two other cases eRMS was diagnosed in a boy at the age of 4 years (ID18) and a girl at the age of 1 year and 6 months (ID30) (16, 24, 33, 50). Also, an eRMS in orbit was presented in patient ID2 at the age of 10 months (44). Bilateral Wilm’s tumor mentioned earlier as renal disease was revealed in patient ID19, and congenital yolk sac tumor was identified in the newborn boy (ID36) (17). In adult group, patients ID31, ID32 and ID50 had a basal cell carcinoma, squamous cell carcinoma, and Bowen’s disease (9, 33). One case of astrocytoma was presented in a 44-years old woman (ID6) (47).

Benign tumors excluding previously described localizations (central nervous, visual and renal systems) were found in 14 (28%) patients (Figure 3). Lesions of skin and mucous included tricholemmoma (4), papilloma (4), syringocystadenoma papilliferum (9, 32), sebaceoma (32), poroma (32) and trichoblastoma (32). The group of non-skin benign neoplasms included thyroid nodule (15, 24, 25), sialoadenoma (35), ganglioneuroma (34), ovarian cyst (24), right lateral canthus soft tissue tumor (31), odontogenic tumors (14), giant-cell tumor of the jaw (33), pineal hamartoma (17), and lymphangioma (11). Patients ID30, ID32, ID36, ID50 had both benign and malignant tumors.

### 3.5 Statistical analysis

Fisher’s exact test was used to search for genotype-phenotype associations between clinical features and the mutations in a specific gene (Table 1).

**TABLE 1:** Genotype-phenotype associations revealed in the study.

Extracutaneous anomalies	*KRAS* mutation (*n* = 30)	*HRAS* mutation (*n* = 17)	OR	95%CI	*P*-value
**CNS**					
Present	25 (83%)	7 (41%)	7.6	2.0–30.3	0.004*
Not found	5 (17%)	10 (59%)			
**Ophtalmic**					
Present	22 (73%)	4 (24%)	8.9	2.2–35.6	0.002*
Not found	8 (27%)	13 (76%)			
**Cardiovascular**					
Present	14 (47%)	5 (29%)	2.1	0.6–7.4	0.35
Not found	16 (53%)	12 (71%)			
**Skeletal**					
Present	8 (27%)	11 (65%)	5.0	1.4–18.1	0.015*
Not found	22 (73%)	6 (35%)			
**Benign tumors**					
Present	7 (23%)	5 (29%)	0.73	0.19–2.8	0.73
Not found	23 (77%)	12 (71%)			
**Malignant tumors**					
Present	4 (13%)	7 (41%)	0.21	0.05–0.91	0.07
Not found	26 (87%)	10 (59%)			

Statistically significant *p*-values are marked by (*).

Patients with *NRAS* mutations were presented only in three cases, so we excluded them from statistical analysis. We also did not include those manifestations that were found in the sample under 20%. Analyzing 50 clinical cases described in the literature, we found that pathological changes in the central nervous system were more common in patients with *KRAS* mutations compared to patients with *HRAS* mutations (83 vs. 41%, OR = 7.6, 95%CI = 2.0–30.3, *p* = 0.004). In addition, patients with *KRAS* mutations were more likely to have ophthalmological lesions than patients with *HRAS* mutations (73 vs. 24%, OR = 8.9, 95%CI = 2.2–35.6, *p* = 0.002). At the same time, patients with *HRAS* mutations were more frequent to develop skeletal bone abnormalities, including hypophosphatemic rickets, than patients with *KRAS* mutations (65 vs. 27%, OR = 5.0, 95%CI = 1.4– 18.1, *p* = 0.015). No association was found between a mutation in a specific gene and cardiovascular malformations or the development of benign or malignant neoplasms of different localizations.

Further stratified analysis based on specific amino acid substitutions in the *KRAS* and *HRAS* genes was performed (Table 2).

**TABLE 2:** Genotype-phenotype associations for specific mutations in the KRAS and HRAS genes.

Extracutaneous anomalies	*KRAS* (*n* = 30)		*HRAS* (*n* = 17)	
	G12D (*n* = 19)	Other *KRAS*-mut (*n* = 11)	G13R (*n* = 10)	Other *HRAS*-mut (*n* = 7)
**CNS**				
Present	16 (84%)	9 (81%)	4 (40%)	3 (43%)
Not found	3 (16%)	2 (19%)	6 (60%)	4 (57%)
G12D vs. Other *KRAS*-mut, OR = 1.01, *p* = 1.00			G13R vs. Other *HRAS*-mut, OR = 1.14, *p* = 1.00	
*KRAS* G12D vs. *HRAS* G13R, OR = 8.0, 95%CI = 1.4–46.8, *p* = 0.03*				
**Ophtalmic**				
Present	14 (74%)	8 (73%)	2 (20%)	3 (43%)
Not found	5 (26%)	3 (27%)	8 (80%)	4 (57%)
G12D vs. Other *KRAS*-mut, OR = 1.01, *p* = 1.00			G13R vs. Other *HRAS*-mut, OR = 0.33, *p* = 0.59	
*KRAS* G12D vs. *HRAS* G13R, OR = 11.2, 95%CI = 1.8–71.7, *p* = 0.016*				
**Cardiovascular**				
Present	9 (47%)	5 (45%)	2 (20%)	4 (57%)
Not found	10 (53%)	6 (55%)	8 (80%)	3 (43%)
G12D vs. Other *KRAS*-mut, OR = 1.08, *p* = 1.00			G13R vs. Other *HRAS*-mut, OR = 0.16, *p* = 0.18	
***KRAS* G12D vs. *HRAS* G13R, OR = 3.6, 95%CI = 0.6–21.6, *p* = 0.23**				
**Skeletal**				
Present	4 (21%)	4 (36%)	7 (70%)	4 (57%)
Not found	15 (79%)	7 (64%)	3 (30%)	3 (43%)
G12D vs. Other *KRAS*-mut, OR = 1.01, *p* = 1.00			G13R vs. Other *HRAS*-mut, OR = 0.22, *p* = 0.28	
*HRAS* G13R vs. *KRAS* G12D, OR = 8.8, 95%CI = 1.5–50.1, *p* = 0.017*				
**Benign tumors**				
Present	5 (26%)	3 (27%)	4 (40%)	1 (14%)
Not found	14 (74%)	8 (73%)	6 (60%)	6 (86%)
G12D vs. Other *KRAS*-mut, OR = 6.6, *p* = 0.26			G13R vs. Other *HRAS*-mut, OR = 4.0, *p* = 0.33	
***KRAS* G12D vs. *HRAS* G13R, OR = 0.53, 95%CI = 0.1–2.7, *p* = 0.67**				
**Malignant tumors**				
Present	4 (21%)	0 (0%)	6 (60%)	1 (14%)
Not found	15 (79%)	11 (100%)	4 (40%)	6 (86%)
G12D vs. Other *KRAS*-mut, OR = 6.7, *p* = 0.27			G13R vs. Other *HRAS*-mut, OR = 9.0, *p* = 0.13	
***HRAS* G13R vs. *KRAS* G12D, OR = 5.6, 95%CI = 1.0–30.1, *p* = 0.051**				
*HRAS* G13R vs. *KRAS-*mut, OR = 9.7, 95%CI = 1.9–50.6, *p* = 0.007*				
*HRAS* G13R vs. other *KRAS*- and *HRAS*-mut, OR = 9.6, 95%CI = 1.9–46.5, *p* = 0.005*				

Other KRAS-mutations include G12V, G12C, G13D, A146, and K117. Other HRAS-mutations are G12S, G12C, G13V, and Q61R. Statistically significant *p*-values are marked by (*).

The impact of the most common *KRAS* G12D and *HRAS* G13R mutations was examined separately, while rarer mutations in the *KRAS* gene (G12V, G12C, G13D, A146, and K117) and in the *HRAS* gene (G12S, G12C, G13V, and Q61R) were combined into the corresponding groups of other mutations. We found no significant differences in the distribution of extracutaneous anomalies within patients with different *KRAS* mutations or *HRAS* mutations. When comparing patients with *KRAS* G12D and *HRAS* G13R, the same patterns were found as when considering the overall patient groups with *KRAS* and *HRAS* mutation (Table 2). Namely, among patients with *KRAS* G12D relative to *HRAS* G13R patients, CNS pathological features were more common (84 vs. 40%, OR = 8.0, 95%CI = 1.4–46.8, *p* = 0.03), as well as ophthalmological manifestations (74 vs. 20%, OR = 11.2, 95%CI = 1.8–71.7, *p* = 0.016). At the same time, skeletal abnormalities were more frequent in patients with *HRAS* G13R compared to patients with *KRAS* G12D (70 vs. 21%, OR = 8.8, 95%CI = 1.5–50.1, *p* = 0.017). No significant differences were found in the distribution of cardiovascular manifestations and the presence of benign tumors. As for malignant neoplasms, various malignant tumors were found in 60% of patients with *HRAS* G13R, which is notably more often than in other patient groups: in other *HRAS*-mut patients it was 14% (*p* = 0.13), in *KRAS* G12D patients 21% (*p* = 0.51), and in the whole *KRAS*–mut group 13 % (OR = 9.7, 95%CI = 1.9–50.6, *p* = 0.007), respectively.

## 4 Discussion

SFMS is a genetic disease characterized by the presence of a nevus sebaceous with involvement of multiple systems of organs (extracutaneous abnormalities). The cause of the disease is somatic mosaicism of the RAS signaling pathway genes, namely *KRAS, HRAS* and *NRAS*.

Nevus sebaceous is a hallmark of SFMS. Histological examination of skin biopsy can reveal an atrophic epidermis, immature hair shafts, prominent smooth muscle bundles and only a few sebaceous glands. Although nevus is a defining feature, it is only one aspect of a broader syndrome, and it can also occur in approximately 0.3% of newborns without other syndromic manifestations (51). The classic SFMS triad includes sebaceous nevus combined with epileptic seizures and intellectual disability (8). However, recent data show that the phenotypic spectrum of SFMS manifestations is highly variable and may affect almost all systems of the human body.

When we analyzed the full texts of selected articles, we noticed that authors used different synonyms of this disease—linear nevus sebaceus syndrome (LNSS), linear sebaceus nevus syndrome (LSNS), nevus sebaceous syndrome (NSS), Schimmelpenning syndrome (SS), Schimmelpenning-Feuerstein-Mims syndrome (SFMS). This created certain difficulties in interpreting the data from the research articles.

In some cases, patients with the SFMS phenotype had a different diagnosis. Due to overlapping clinical presentations and the identification of specific symptoms, patients ID14, ID24, ID30, ID31, ID38, ID40, and ID41 were diagnosed with cutaneous skeletal hypophosphatemic syndrome (CSHS). This disease is characterized by the presence of congenital nevi, rickets, hypophosphatemia, and elevated levels of fibroblast growth factor FGF23 (15, 20, 33, 43, 49), therefore it includes individual cases of SFMS, but also other diseases. Depending on the type of congenital nevus (melanocytic, keratinocytic, etc.), the CSHS group may include patients with phacomatosis pigmentokeratotica and other diseases, if hypophosphatemic rickets is present as a clinical manifestation. In this regard, it is extremely important to clarify the histological type of nevus, as well as the presence of concomitant clinical manifestations, in order to establish an accurate diagnosis. We included patients ID14, ID24, ID30, ID31, ID38, ID40, and ID41 in the study because they had linear epidermal nevi combined with cardiovascular, neurological, and ophthalmological disorders, as well as benign tumors, which corresponded to the clinical presentation of SFMS.

Patients ID4, ID8, and ID42 were diagnosed with epidermal nevus syndrome as the main diagnosis (34, 45, 48). Epidermal nevus syndrome (ENS) includes a number of diseases, namely: SFMS, phakomatosis pigmentokeratotica, CHILD syndrome (OMIM #308050), comedone nevus syndrome (OMIM #617025), Becker nevus syndrome (OMIM #604919), etc. These diseases differ in both their molecular genetic mechanisms of development and their clinical manifestations. After a detailed analysis of the described phenotypes, patients ID4, ID8, ID42 were classified as SFMS based on genetic testing results and specific phenotypes. The same can be observed in patients ID19, ID21-23, and ID49, who were diagnosed with mosaic or somatic RAS pathology without further details (23, 24, 50).

Accordingly, molecular genetic testing is extremely important for refining the diagnosis of SFMS. In most cases, known hotspot mutations have been detected in the *KRAS*, *HRAS*, or *NRAS* genes (12, 13, or 61 codons). Activating mutations in the *RAS* family genes cause the formation of a GTP-bound active form of the protein, which leads to the hyperactivation of a number of intracellular signaling pathways that control cell growth and proliferation. The predominant mutation in the *KRAS* gene was G12D (63%), and in the *HRAS* gene, it was G13R (59%). Rarer genetic variants, such as *KRAS* c.351A > C (p.Lys117Asn, K117N) (7) and c.436G > A, (p.Ala146Thr, A146T) (28, 42), identified in patients with SFMS, have also been previously described as mutations associated with the actively proliferating cell phenotype (52).

However, a comparative analysis of the activating effects of *KRAS* mutations affecting codons 12, 13, 61, 117, and 146 showed that different mutations can have different effects on *KRAS* activity, signal transduction, and cellular transcription programs. In particular, *KRAS* exon 4 mutations K117 and A146 were associated with lower levels of GTP-bound RAS protein (53, 54). It has previously been noted that in patients with somatic mosaicism, the type of mutation can also influence the character of clinical manifestations. Thus, a mosaic *KRAS* c.436G > A, (A146T) mutation, identified in three patients with SFMS (28, 42), has also been described in a number of patients with other similar syndromes, such as oculoectodermal syndrome (OES, OMIM #600268) and encephalocraniocutaneous lipomatosis (ECCL, OMIM #613001), which are characterized by a range of interconnected anomalies such as scalp lesions, epilepsy, epibulbar dermoid, cloudy cornea, eyelid coloboma, aorta coarctation, and variations in skin pigmentation.

Somatic mutations in the *KRAS*, *HRAS*, and *NRAS* genes are often presented in different cancers. At the same time, mutations in the *KRAS* gene are most often found in pancreatic carcinoma, colorectal and lung cancer; mutations in the *HRAS* gene commonly diagnosed in skin cancer and tumors of the head and neck (especially rhabdomyosarcoma); and *NRAS* mutations can be detected in melanoma and hematological malignancies (55). In our study, benign or malignant tumors were found in 22 cases (44%), among them four patients had both benign and malignant neoplasms. No associations were revealed between mutation in a specific gene and the development of benign tumors. At the same time, patients with the *HRAS* G13R mutation developed malignant neoplasms much more frequently than other patient groups. The most common diagnosis was skin cancer (basal cell carcinoma or squamous cell carcinoma), then rhabdomyosarcoma, and astrocytoma.

It is generally accepted that the extreme phenotypic variability typical of mosaic disorders including SFMS, is based on the timing of mutation development, which affects its distribution throughout the body, while the influence of a specific gene has not been studied in detail. At the same time, based on the analysis of 50 clinical cases, we found that patients with mosaic mutations in the *KRAS* gene were significantly more likely to develop central nervous system abnormalities and ophthalmological disorders compared to *HRAS*-mut patients. Also, renal diseases were only observed in patients with *KRAS* mutations, while the development of skeletal abnormalities was significantly associated with mutations in the *HRAS* gene. We found that the different *KRAS* or *HRAS* mutations involved in the development of these disorders acted in the same way, and similar patterns were also found when comparing the range of clinical manifestations in groups with *KRAS* G12D and *HRAS* G13R mutations. These issues certainly require further study, involving a larger number of genetically confirmed SFMS cases.

On the other hand, we believe that this may indicate a fine-tuning of mechanism for implementation of genetic information, including various levels of expression of *RAS* family genes in different organs and tissues during embryonic development. Thus, a mouse model has shown that at the embryonic stage, the KRAS protein is expressed in a wide range of organs and tissues, but near birth it becomes restricted to specific cell types (56), thus activating *KRAS* mutations may have various effects in different tissues and organs. It was shown, for example, that *KRAS* G12D expression in lymphatic endothelial cells during embryonic development impaired the formation of lymphovenous valves and caused the enlargement of lymphatic vessels (57).

There are also a number of studies concerning the involvement of the *HRAS* gene in bone homeostasis by controlling osteoblast differentiation, adaptation to mechanical strain and repression of RANKL expression in mature osteoblasts, the cells responsible for building new bone (58). A recent study in mouse cell lines demonstrated that the activating mutations p.Gly12Ser (G12S) and p.Gly12Val (G12V) in the *HRAS* gene increased the expression of osteopontin (OPN), which inhibited the differentiation of osteoblasts, with the pathogenic variant G12V having a more pronounced inhibitory effect (59). Further research in this direction will allow understanding the association of *HRAS* mutations with the development of skeletal anomalies in SFMS syndrome.

In the articles we analyzed, the proportion of the mutant allele in the affected tissues ranged from 3 to 73%, while the mutation was not detected in peripheral blood and healthy skin samples. This once again emphasizes that in diseases associated with somatic mosaicism, diagnosis should be carried out on affected tissues (nevi, tumors). At the same time, it is recommended to use high-tech research methods (NGS) to determine low-abundant mutant alleles. Identification of the gene involved in the pathogenesis of SFMS and the type of mutation can be used not only to confirm the diagnosis, but also to further predict the development of individual clinical manifestations and to develop an individual monitoring program.

## 5 Conclusion

The most common disorders in patients with SFMS were CNS, ophthalmological, and cardiovascular anomalies, as well as skeletal lesions, which occurred in 68, 56, 44, and 42% of cases, respectively. Based on the obtained data, two clinical and genetic groups of patients with SFMS can be distinguished: *KRAS-mut* and *HRAS-mut*. Most frequent mutations in these groups were *KRAS* G12D and *HRAS* G13R. The patients with pathogenic variants in the *KRAS* gene most often suffered from damage to the organs of vision, central nervous system, and kidneys. Hearing loss was also frequently detected in this group. At the same time, the presence of a mutation in the *HRAS* gene most often caused bone abnormalities, including hypophosphatemic rickets. In addition, reproductive system was affected mainly in *HRAS*-mut patients, while malignant tumors were found mostly in patients with *HRAS* G13R mutation. Patients with *NRAS* mutations were presented only in three cases and all of them had a mutation Q61R. Patients with *NRAS-*mut had bone abnormalities (hypophosphatemic rickets), central nervous system disorders, and ocular abnormalities at the same frequency. No one had a precocious puberty or malignant tumor. Thus, this group intermediates between *KRAS*-mut and *HRAS*-mut patients, but due to the extremely small sample size, it is difficult to draw definitive conclusions.

Regardless of the gene involved, patients with SFMS had an increased risk of developing reproductive system disorders (8%), benign tumors (28%), and malignant neoplasms (22%), even in early childhood. In 7 out of 11 patients, these manifestations appeared before the age of 5 years, which deserves special attention. Based on the data obtained, a dynamic follow-up plan for patients may be recommended, which should include annual consultations with a dermatologist, neurologist, ophthalmologist, cardiologist, endocrinologist, and oncologist, MRI of the brain, ultrasound of the abdomen and kidneys, as well as an electroencephalogram in the presence of seizure syndrome, X-ray, and densitometry with determination of biochemical indicators of bone tissue condition. Such an examination plan will allow for the timely detection of possible disease and the determination of optimal measures for their correction.

## 6 Limitations and strengths

Our study presents the first systematic review summarizing the available clinical data on genetically confirmed SFMS through case reports and case series. The use of precise diagnostic criteria in the selection of articles allowed us to identify correlations between genotype and phenotype for such a complex and clinically variable disease as SFM syndrome. However, this systematic review has several limitations. The first limitation was the small number of study participants. On the one hand, this may be due to the rarity of SFMS and the difficulties in diagnosing this disease. On the other hand, not all patients with this syndrome underwent genetic testing. In addition, in some cases, only the patient’s blood (leukocytes) was sent to the laboratory, and the affected tissue (e.g., nevus) was not tested. For all of the above reasons, a large group of patients with SFMS was excluded from this study due to the lack of relevant DNA test results. The second limitation was that 13 patients were younger than 1 year at the time of publication, so the manifestation of some clinical signs, such as premature or delayed sexual development, hypophosphatemic rickets, epilepsy, or tumors (especially skin cancer) might have been detected in an older age group. The third limitation was the lack of long-term follow-up data in most cases, which made it difficult to assess the development of new symptoms. The fourth limitation was the lack of data on comprehensive patient examinations. In this regard, it was difficult to determine whether patients did not have certain signs or whether they had not undergone the necessary examinations to detect them. The fifth limitation that needs to be mentioned is the potential risk of publication bias, as case reports with more severe phenotypes are likely to be published.

## Data Availability

The original contributions presented in the study are included in this article/Supplementary material, further inquiries can be directed to this corresponding author.
