# Supplementary figures and images for: Schimmelpenning-Feuerstein-Mims syndrome: a systematic review of clinical cases to identify genotype-phenotype associations
Source: Front Med (Lausanne). 2025 Dec 8;12:1681584. doi: 10.3389/fmed.2025.1681584 (PMC12719304; doi:10.3389/fmed.2025.1681584)

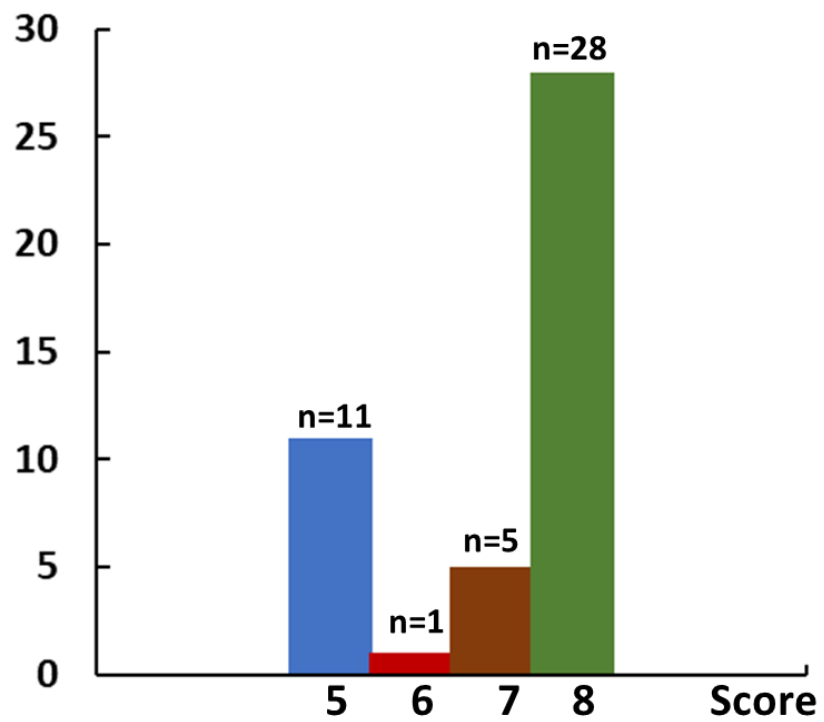

Figure S1. The summary of JBI quality assesement using JBI Checklist for Case Reports.

Supplement: Supplementary file 1 [file Data_Sheet_1.pdf]
