# Supplementary material for: Schimmelpenning-Feuerstein-Mims syndrome: a systematic review of clinical cases to identify genotype-phenotype associations
Source: Front Med (Lausanne). 2025 Dec 8;12:1681584. doi: 10.3389/fmed.2025.1681584 (PMC12719304; doi:10.3389/fmed.2025.1681584)
Supplement: Supplementary file 4 [file Table_3.docx]

Table S3. JBI Checklist for systematic reviews

| № | Question | Answer |
| --- | --- | --- |
| 1 | Is the review question clearly and explicitly stated? | Yes  *(Finding correlation between genetic changes and clinical presentation among the patients with SFMS for following observation according to individual risk)* |
| 2 | Were the inclusion criteria appropriate for the review question? | Yes  *(Only patients with SFMS and genetic testing were included in this review)* |
| 3 | Was the search strategy appropriate? | Yes  *(Search strategy was made using PRISMA)* |
| 4 | Were the sources and resources used to search for studies adequate? | Yes  *(The sources used to search for studies were official open databases and one of the biggest in medical scientific resources)* |
| 5 | Were the criteria for appraising studies appropriate? | Yes  *(All articles were checked using JBI checklist for clinical cases and second check with our own checklist)* |
| 6 | Was critical appraisal conducted by two or more reviewers independently? | Yes  *(Two researcher independently searched the articles)* |
| 7 | Were there methods to minimize errors in data extraction? | Yes  *(First stage of searching included wide spectrum of keywords for avoiding loss of article with synonymic name of disease. Also we used our own checklist for check described patients and revealed just patients with SFMS)* |
| 8 | Were the methods used to combine studies appropriate? | Yes  *(It included the description and statistical processing of the received data)* |
| 9 | Was the likelihood of publication bias assessed? | Yes  *(Statistical analysis was performed)* |
| 10 | Were recommendations for policy and/or practice supported by the reported data?* | Yes  *(Common recommendations for patients with SFMS did nott exist because of low frequency of this syndrome; reported data correlated with recommendation by single medical groups from different countries )* |
| 11 | Were the specific directives for new research appropriate?* | Yes  *(The authors discussed fundamental issues concerning the influence of genes on tissue formation during embryogenesis)* |
